# Eurasian jays (*Garrulus glandarius*) show episodic-like memory through the incidental encoding of information

**DOI:** 10.1371/journal.pone.0301298

**Published:** 2024-05-15

**Authors:** James R. Davies, Elias Garcia-Pelegrin, Nicola S. Clayton

**Affiliations:** 1 Department of Psychology, University of Cambridge, Cambridge, United Kingdom; 2 Department of Psychology, National University of Singapore, Singapore, Singapore; University of Girona School of Education and Psychology: Universitat de Girona Facultat d’Educacio i Psicologia, SPAIN

## Abstract

Episodic memory describes the conscious reimagining of our memories and is often considered to be a uniquely human ability. As these phenomenological components are embedded within its definition, major issues arise when investigating the presence of episodic memory in non-human animals. Importantly, however, when we as humans recall a specific experience, we may remember details from that experience that were inconsequential to our needs, thoughts, or desires at that time. This ‘incidental’ information is nevertheless encoded automatically as part of the memory and is subsequently recalled within a holistic representation of the event. The incidental encoding and unexpected question paradigm represents this characteristic feature of human episodic memory and can be employed to investigate memory recall in non-human animals. However, without evidence for the associated phenomenology during recall, this type of memory is termed ‘episodic*-like* memory’. Using this approach, we tested seven Eurasian jays (*Garrulus glandarius*) on their ability to use incidental visual information (associated with observed experimenter made ‘caches’) to solve an unexpected memory test. The birds performed above chance levels, suggesting that Eurasian jays can encode, retain, recall, and access incidental visual information within a remembered event, which is an ability indicative of episodic memory in humans.

## Introduction

The ability to journey through one’s own subjective time, traveling backwards to remembered events as well as forwards to possible future events, is referred to as mental time travel. When looking backwards and consciously reconstructing personally experienced events, we engage in the form of memory known as episodic memory [1–3]. As such, recalling the past is often differentiated between ‘knowing’ vs ‘remembering’ [1], with episodic memory representing the latter. In contrast, *semantic* memory concerns the recall of factual information with no associated conscious experience, and so can be thought of as simply ‘knowing’. Together, episodic memory and semantic memory make up the declarative, or explicit, memory system which concerns the encoding and storage of long-term memories that can be actively accessed [1,2].

As the major distinction between semantic and episodic memory is that episodic memory involves a conscious experience during recall, its characteristic phenomenology is intrinsic within its definition [3–5]. This results in major issues when investigating the presence of episodic memory in non-human animals, as evidence for conscious episodic recall in humans is centred around language-based reports and there are currently no agreed upon non-linguistic behavioural markers of consciousness [6]. Whilst many psychologists believe episodic memory (and thus mental time travel) to be a uniquely human ability [3,7–10], arguing that although animals have a complex semantic knowledge of their environment they cannot consciously recall and re-experience past events as we can [8], it is theoretically impossible to establish if non-humans have episodic memory using this definition. Consequently, researchers have instead focused on behavioural paradigms that represent the characteristics of human episodic memory in non-human animals [11–24]. However, in the absence of evidence for a subjective conscious experience during recall, this type of memory is termed ‘episodic-like memory’ [11].

In pioneering research on episodic-like memory, Clayton and colleagues [11–13,25,26] conducted a sequence of important experiments with scrub-jays (*Aphelocoma sp*.). As these jays cache long-lasting foods (e.g., nuts) and quickly perishing foods (e.g., insect larvae) for future consumption, it is argued that episodic-like memory has evolved in these birds to facilitate the remembering of the contents, location, and timing of their caches, and therefore the ability to recover these foods before they become inedible [27]. By exploiting this natural phenomenon, these studies demonstrate that jays, when recovering trial-unique caches after varying time intervals, are recalling integrated representations of the ‘what’ (food type), ‘where’ (cache location), and ‘when’ (time of caching relative to retrieval) information and use these memories flexibly to dictate behaviour [24]. Since these seminal studies, other researchers have used the ‘what-where-when’ paradigm to test species across taxa, including rodents [28–32], great apes [33], other corvids [14], parids [34], and cuttlefish [35].

However, it has been suggested that subjects in these experiments could have been successful through the use of non-episodic mechanisms, and thus without necessarily recalling the encoding event in an episodic-like manner [15–19,23,36]. As this paradigm requires repeated training to learn the experimental rules (such as the degradation rates of certain foods), the animals may learn to expect an upcoming memory test after the presentation of the encoding situation. Therefore, they may learn that some specific information within the presented event is required to use in the expected upcoming memory test. Subsequently, the animal may be able to retain and carry forward a memory trace representing this information in order to use later in the anticipated memory assessment, without actually remembering back to the original event *upon* presentation of this memory test (as is required for an episodic-like memory account) [37].

An alternative paradigm used to test episodic-like memory in non-human animals, and arguably the most convincing [37,38], is the incidental encoding and unexpected question paradigm [15]. The rationale behind this paradigm is that when we, as humans, engage in episodic memory and recall a specific experience, we may remember details from that event that were inconsequential to our needs, thoughts, or desires at that time. This trivial, or ‘incidental’, information is nevertheless encoded automatically as part of the memory [39] and is subsequently recalled as part of a holistic representation of the event. For example, we may recall incidental visual information when remembering a visit to the supermarket the day before, such as the colour of the cashier’s t-shirt or the pattern on the countertop. This paradigm thus aims to behaviourally represent the incidental encoding characteristic of human episodic memory, by ‘unexpectedly’ asking subjects to recall incidental information about a specific event. As these tests are unexpected to the subjects, they do not learn that any specific information within an encoding trial will later become relevant for an upcoming memory test. If subjects are able to use information, that was irrelevant to solving an encoding trial to obtain success in a subsequent memory test, this demonstrates that they are able to encode, recall and access incidental information within a remembered event, which is a capability characteristic of episodic memory in humans [23]. Researchers have used the incidental encoding and unexpected question paradigm to investigate episodic-like memory in various non-human taxa [15–23].

Whilst episodic-like memory has been extensively studied in a few corvid species using the what-where-when memory paradigm [11–14,25,26], to our knowledge no researchers have assessed their abilities using the incidental encoding and unexpected question paradigm. Consequently, we tested Eurasian jays (*Garrulus glandarius*), a member of the Corvidae family and relative of the scrub-jay, on their ability to use incidentally encoded information in an unexpected memory task. Like scrub-jays, Eurasian jays habitually cache both perishable and non-perishable foods for later consumption and depend heavily on these caches to sustain them through periods of low food availability [40]. Furthermore, they show evidence for complex cognitive abilities, such as object permanence [41], observational spatial memory [42], and some evidence for the flexible deployment of various cache-protection strategies [43–45] (although these findings were not replicated in later work [46]). Most importantly, however, Eurasian jays have also shown evidence for planning for the future, by overcoming their current desires in order to plan for anticipated future needs [47]. As, in humans, episodic memory is thought to provide the basis from which future orientated thoughts and predictions can be made [48–50], we predicted that Eurasian jays would be able to encode incidental information related to an event, and then subsequently recall this information in order to solve an unexpected memory task.

## Methods

### Subjects and housing

Seven Eurasian jays (3 females, 4 males) participated in this study. The sample was made up of all birds that were available and motivated for testing. All the jays were hand-reared in 2015 and were socially housed within a large outdoor aviary (approximately 20m long × 10m wide × 3m high) at the Sub-Department of Animal Behaviour, University of Cambridge, Cambridgeshire, UK. The aviary was divided at one end into smaller sections (approximately 6 × 2 × 3m) which connected to indoor testing compartments (each 2 x 1 x 2m). The jays were fed a maintenance diet of soaked cat food biscuits, eggs, vegetables, seeds, and fresh fruit and had *ad libitum* access to water (including during testing). To increase their motivation to participate in experiments, the jays’ maintenance diet was removed from the aviary 1-hour before testing. Subjects participated on a voluntary basis (maximising motivation) and were individually separated once they entered the testing compartments. The experimenter interacted with the jays via an open window adjacent to the indoor compartments.

### Procedures

**Fig 1:**
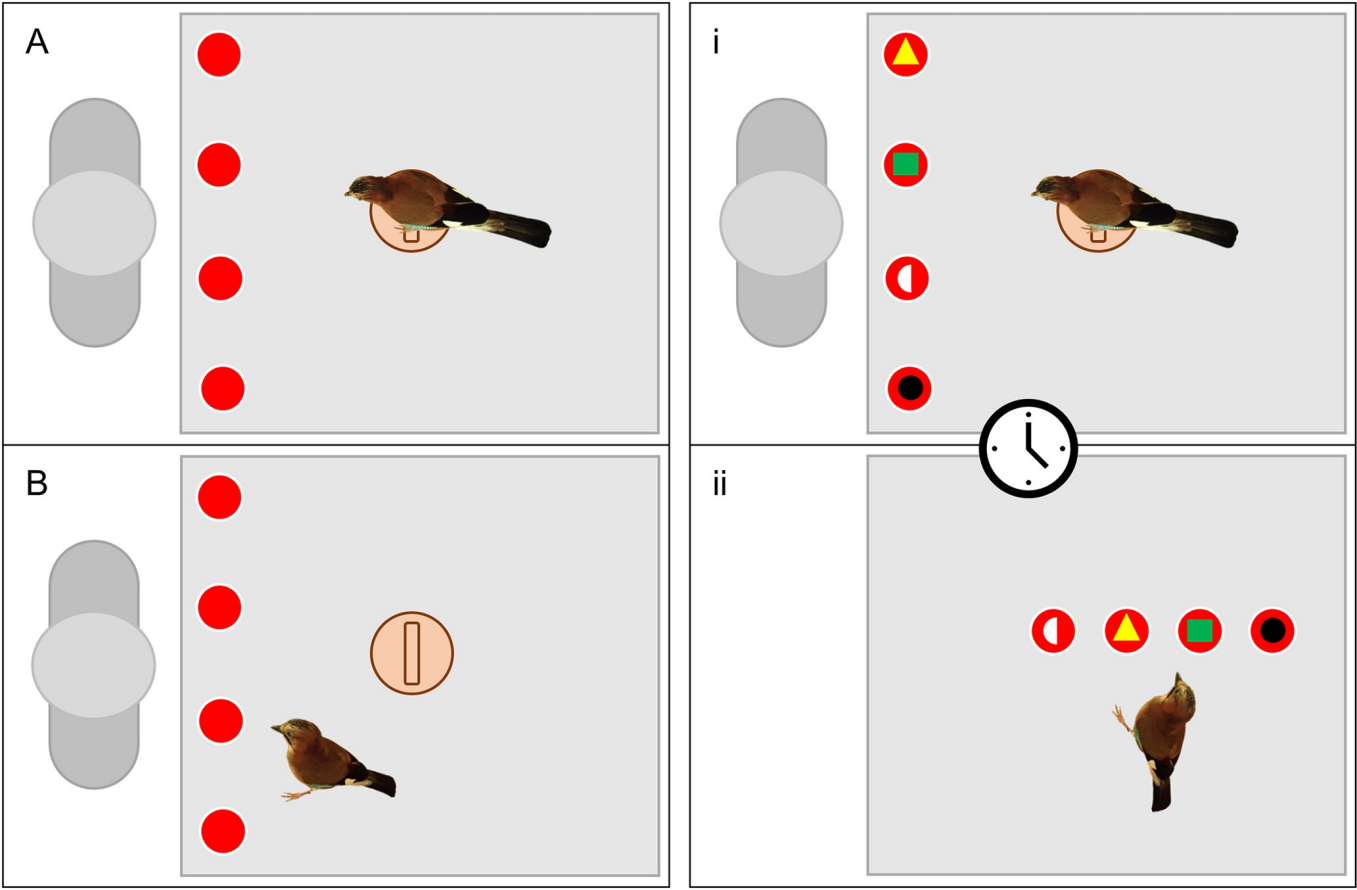


The experiments were reviewed and approved by the University of Cambridge Animal Welfare Ethical Review Body and were conducted under a non-regulated procedure license (NR2021/49). All procedures were non-invasive, purely behavioural and did not require anaesthesia or euthanasia of any subjects. The jays had previous training using cups in previous studies (e.g., [51]). The cups in this study, and other unpublished studies, were always identical and plain red with no other salient and distinctive visual features, as the cups in these experiments simply represented spatial locations that food could be hidden under. Therefore, the jays had no experience with being trained to attend to any visual features (unique or not) on the cups before the onset of this experiment. To confirm their ability to locate food hidden under the cups, a spatial memory training phase was conducted. In this phase, the testing compartment was set up with a platform next to the experimenter window, with a perch placed at the centre. Four identical cups, equally spaced, were arranged in front the perch, parallel to the experimenter window (Fig 1A). The bird entered the compartment and was separated from the rest of the aviary; now only having access to the test compartment and an adjacent closed outside section of the aviary. Whilst remaining on the perch and facing the experimenter, the jays observed as the experimenter turned one of the cups over, placed a mealworm inside, then returned it to its original position (Fig 1A). The birds then made a choice by pulling on a string attached to the top of the cup (S1 Fig) to reveal its contents (Fig 1B). Once a choice was made the trial ended, and the bird was encouraged to return to the perch before the procedure was repeated for a subsequent trial. After baiting a cup, the experimenter orientated their head and eyes directly forward and kept their arms by their side in order to limit the possibility of accidental cues directing the bird’s choice. The position of the baited cup was pseudorandomised, in that no single cup contained the mealworm more than twice in a row. All birds reached training criterion (8/10 successful trials) in a single session of 10 trials.

The set up and procedure of the test phase was almost identical to the training phase, except for the cups used and the addition of an unexpected delayed long-term memory assessment. Instead of identical cups, the cups used in test trials each possessed a unique visual marker as part of an array of different visual elements: either a) coloured card around the string attached to the top of the cup (S1A Fig); b) a laminated coloured shape attached to the front of the cup (S1B Fig); or c) a laminated coloured and/or patterned card underneath the cup (S1C Fig). These visual characteristics were not present during training, and thus did not represent relevant information associated with solving the original trained task. Each bird received a single trial per trial type (string, shape, or card), each on different days separated by at least 24 hours. The order of trials each bird received was pseudorandomised (so that the position of each trial type in the sequence was counterbalanced across individuals) and unique (except Stuka and Booster; S1 Table). One bird, Sojka, failed to make a choice in her string trial within 15 minutes after entering the test compartment and so was excluded from this trial type. Immediately before each test trial, a minimum of 5 and a maximum of 10 trials (identical to the training procedure but with the test cups) were conducted to ensure the jays were still performing accurately with spatial memory and not failing due to other factors unrelated to the study (e.g., fear, lack of motivation, etc.). As no cup was baited more than the others, and the bird had immediate access to recover the reward underneath the cup, the visual characteristics of the cups were again not relevant to solve the task at this stage. As with the training phase, the only information necessary to solve this task was spatial information, meaning the birds only had to use short-term spatial working memory to be successful. Once an individual reached 5 consecutive correct re-training trials, a single test trial was conducted. The first stage of this trial (Fig 1i) was identical to the training procedure, except that 5 mealworms were used to bait the cup (to increase the saliency of the caching event) and the bird was prevented from reaching the cups to prematurely make a choice (a plastic bar was placed over the cups). Once the cup was baited, the bird was removed from the testing compartment (into the outdoor section) and was left alone for 10 minutes without visual access to the cups or the testing compartment. Concurrently, the experimenter moved the cups into a new, distinct location (perpendicular to the test window) and positioned them at random (Fig 1ii). The mealworms were removed from under the cups to control for any visual, auditory, or olfactory cues, thus forcing the birds to rely on memory alone in the succeeding test. Once 10 minutes passed, the bird was brought back into the test compartment and allowed to make a choice. The compartment doors were set up so that the birds faced the cups from the front, and thus were roughly at an equal distance from the jay upon their presentation.

As the visual markers were present on the cups during the re-training trials before each test trial, the birds could potentially use two different strategies to solve this task: 1) rely on the spatial information, or 2) rely on the visual information (or a combination of both strategies). Whilst the birds were exclusively trained to use spatial information to solve the training task, although unlikely, the use of the second strategy cannot be ruled out using this methodology alone. Therefore, an additional control task was conducted to determine whether the birds prioritised spatial information over visual and whether they would learn to use visual information when spatial information was no longer relevant, across the same number of trials as the retraining minimum (*n* = 5). This task was identical to the re-training trials, except that before the bird was allowed to make a choice, the cups were quickly rearranged out of sight (behind a visual occluder). This way, the original spatial cues were still present (i.e., there was a cup in the same location as before) but now the use of the first strategy would lead to failure. The position of each cup after rearranging was pseudorandomised so that the baited cup was never in the same spatial position as it was previously. Crucially, if the jay chose the cup with the same visual marker (rather than in the same position), they received the reward underneath it, thus allowing them the opportunity to learn to solve the task using visual information. Each of the 7 jays performed this task following the main testing period, and the visual markers used (trial type) were counterbalanced across subjects (with an extra session of ‘shape’).

### Analysis

All analysis was conducted using RStudio [52]. We conducted binomial generalised linear mixed models (GLMMs) to investigate whether the jays chose the correct cup above chance levels (0.25), with ‘individual’ as a random effect, for both the main and control test data. To test against the null value of 0.25, we included an offset model (the logit transform of the null value) as a fixed effect. An additional binomial generalised linear model (GLM) was run to assess if the experimental factors had an effect on the birds’ performance in the main test, with ‘condition’ (string, shape, or card) and ‘trial number’ (the position in the test sequence, i.e., 1, 2 or 3) as fixed effects, including an interaction between these effects. To check our models’ assumptions, we used the DHARMa package [53]. The models did not fail to converge and model assumption checks showed no deviation from expected distributions.

## Results and discussion

**Fig 2:**
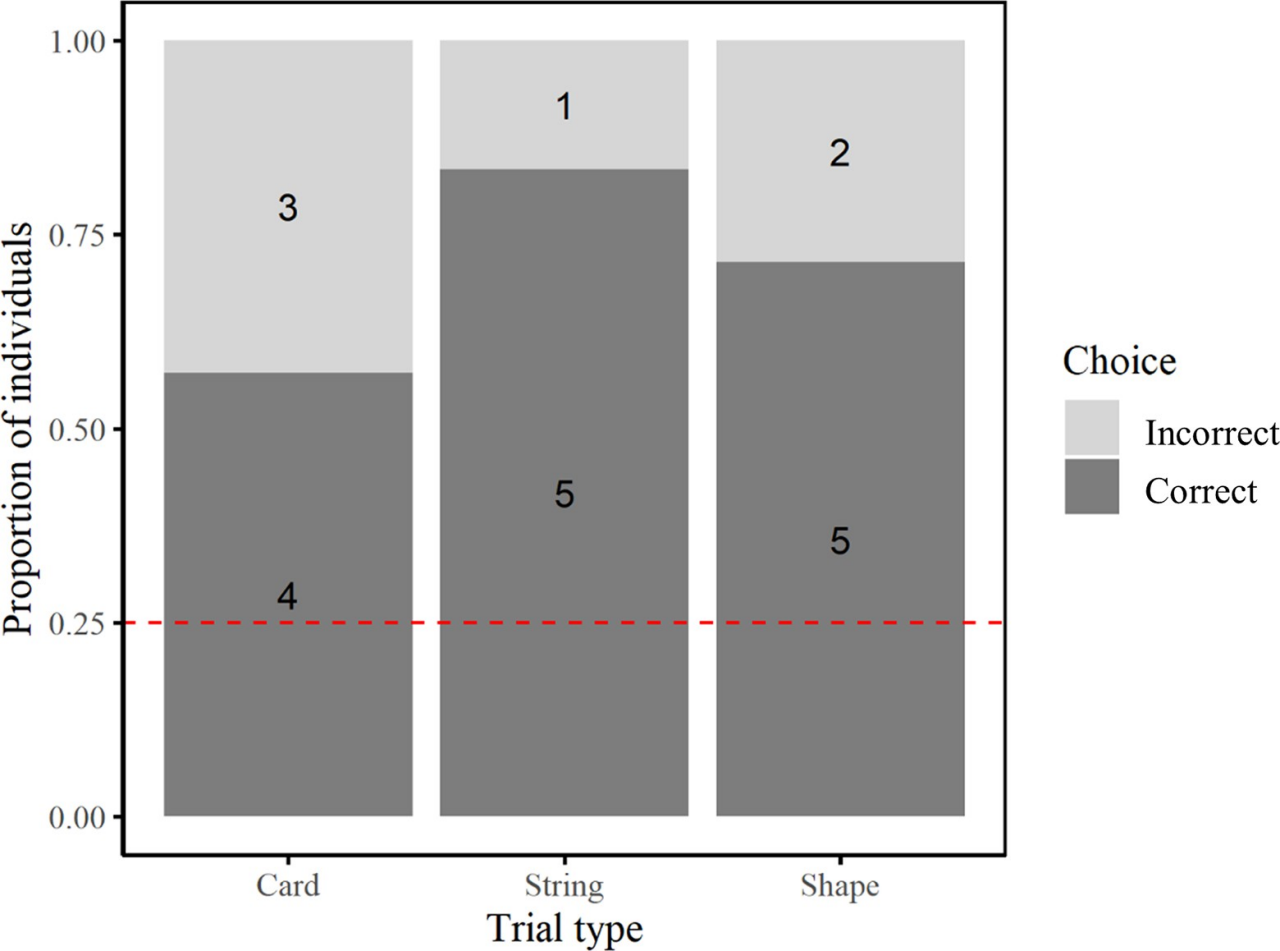


The jays chose the correct cup above chance levels in the main test (binomial GLMM, *p* < 0.001; Fig 2) and there was no significant effect of condition (binomial GLM, *F*_2,14_ = 0.334, *p* = 0.721; Fig 2) or trial number (binomial GLM, *F*_1,14_ = 0.476, *p* = 0.501) on performance, and no significant interaction between these two factors (binomial GLM, *F*_2,14_ = 0.088, *p* = 0.916). In the control test, the still jays chose the correct cup above chance levels according to spatial information, (binomial GLMM, *p* < 0.001; S2 Table), but not according to visual information (binomial GLMM, *p* = 0.151; S2 Table). As the jays showed a success rate significantly above chance levels when selecting a cup in the main test memory phase, this suggests that, in line with our prediction, they were able to encode incidental information within their memories of the original event during the encoding phase and utilise this information upon the unexpected re-presentation of the cups. Furthermore, as they failed to override the use of spatial information in the control task, this finding suggests that they were not actively encoding the visual information regarding the cups in the main test. Taken together, these results demonstrate evidence for episodic-like memory in Eurasian jays.

Whilst multiple studies have provided evidence in support of other corvids possessing an episodic-like memory system [11–14], this is the first such evidence in Eurasian jays. Like other corvids, Eurasian jays habitually cache food in order to sustain them in periods of lower food availability [40]. Therefore, the temporal characteristics involved in this behaviour may have selected for the evolution of an episodic-like memory ability [27]. Amongst other corvids, Eurasian jays are especially reliant on cached food for survival [40], suggesting that this selection pressure may have been comparatively strong in their lineage. Furthermore, Eurasian jays frequently pilfer (steal) conspecific caches, which most closely resembles the experimental situations, as in testing the jays did not cache themselves but instead watched an experimenter cache. To facilitate pilfering, they possess the ability for observational spatial memory [42] and it has been suggested that a species dependency on stored food correlates with their skill at observational spatial memory [36,54]. Moreover, Eurasian jays have been demonstrated to use visual information to locate conspecific caches [42] and seem to, at least in some instances, limit visual information available to competitors when caching themselves [44,45] (but see [46]).

It is important to note, however, that as the birds in the current study were trained to observe the cup bating event and subsequently use this information to select a cup, an immediate test after baiting likely became expected over training. Whilst, at test, a substantial delay occurred between the baiting event and cup selection, the birds still learned to expect a test at the time of baiting, and therefore the encoding of the relevant information (spatial locations) was likely to be explicit. What is important here, however, is that even if the memory test after the delay was somewhat expected, the nature of the test, i.e., the relevant information and long-term storage required to solve it, was unknown to the jays.

Therefore, in order to be successful in this task the jays had to use visual information related to the cups that, at the time of encoding, held no value or relevance to the events unfolding at that time. In the training phase (and the encoding trials), the only information necessary to solve the task and find the food was the spatial position of each cup. Furthermore, this information only needed to be retained for a very short interval as, in this phase, they were allowed to choose a cup almost immediately after the food was hidden. Therefore, only short-term spatial working memory was needed to be successful at this stage of the experiment. However, in the memory phase, after a delay (without visual access to the cups) and once the spatial information no longer existed (as the cups had been moved to distinct locations), the jays had to recall the original event, including the visual characteristics of the cups, in order to solve the task. Whilst these characteristics were present during the short retraining period, meaning there is a possibility that they could have been attended to, without repeated training to specifically learn that these visual characteristics are valuable as cues associated with food (especially when the highly reinforced spatial cues were still available), and without the anticipation of a related memory test for these details, this information was likely to have been encoded incidentally. Indeed, across the same number of trials in the control task the birds did not learn to use visual information when the spatial cues were still present, but were now irrelevant, further suggesting that the visual information of the cups was not explicitly encoded in the main task. The birds’ success in the main task, and failure in the control task, therefore demonstrates evidence for the use of episodic-like memory.

Whilst tests of episodic-like memory are classically designed to ignore the question of consciousness associated with memory recall, success in this paradigm may shed some light on the contents of a non-human animal’s subjective experience. To be successful in the current task, the individual must recall a holistic representation of an event and then target specific incidental information within it in order to solve the unexpected test. Furthermore, recent evidence demonstrates that humans have conscious access to incidentally encoded information within memories and can target this information in order to solve memory tasks [55–58].

It must be said, however, that whilst this study does provide convincing evidence to suggest that Eurasian jays can encode, retain, recall, and access incidental visual information within the remembered event, this ability may be restricted to information associated with food caching, and thus does not necessarily represent the domain-general flexibility typical of human episodic memory [59]. As to our knowledge all research investigating episodic-like memory in corvids relies on some form of food caching paradigm, future studies should develop this work by assessing these birds’ ability to recall other information, such as social cues, as comparable studies have done with other taxa [23].

## Supporting information

S1 Fig Depiction of the unique visual markers: in A) ‘string’ trials (coloured card around the string attached to the top of the cup); B) ‘shape’ trials (a laminated coloured shape attached to the front of the cup); and C) ‘card’ trials (a laminated coloured and/or patterned card underneath the cup).
(DOCX)

S1 Table Summary of test results showing individual choices for each trial, including trial number, trial type (shape, string, and card), the number of retraining trials (*RT*) conducted (until 5 in a row correct) and the arrangement in which the cups were presented in both the encoding phase and the memory phase. Letters (e.g., BWGY) represent the main characteristic colour (B = blue, W = white, G = green, Bk = black, P = pink, and Y = yellow) and the order (from left to right) of the cups. Underlined letters represent the baited cup and therefore the correct choice.
(DOCX)

S2 Table Summary of control test results showing individual choices for each trial, including trial type (shape, string, and card), trial number, and the arrangement in which the cups were presented in both the initial phase and the second phase after rearranging. Letters (e.g., BWGY) represent the main characteristic colour (B = blue, W = white, G = green, Bk = black, P = pink, and Y = yellow) and the order (from left to right) of the cups. Underlined letters represent the baited cup, and thus the correct choice according to the visual information (the cups’ visual features).
(DOCX)
