# Supplementary material for: Eurasian jays (Garrulus glandarius) show episodic-like memory through the incidental encoding of information
Source: PLoS One. 2024 May 15;19(5):e0301298. doi: 10.1371/journal.pone.0301298 (PMC11095760; doi:10.1371/journal.pone.0301298)

**Eurasian jays (*Garrulus glandarius*) show episodic-like memory through the incidental encoding of information**

**James R. Davies, Elias Garcia-Pelegrin, and Nicola S. Clayton**

**S1 Fig.** Depiction of the unique visual markers: in **A**) ‘string’ trials (coloured card around the string attached to the top of the cup); **B**) ‘shape’ trials (a laminated coloured shape attached to the front of the cup); and **C**) ‘card’ trials (a laminated coloured and/or patterned card underneath the cup).


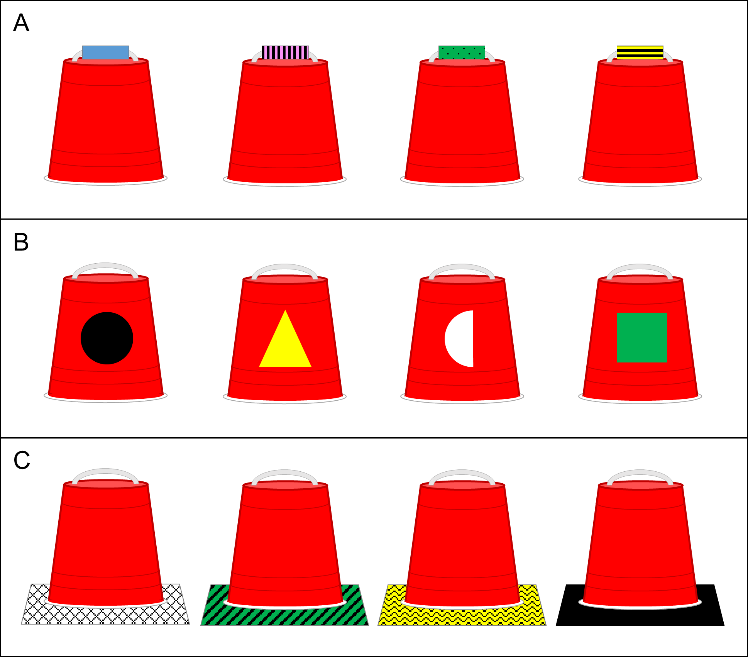

Supplement: S1 Fig — Depiction of the unique visual markers: in A) ‘string’ trials (coloured card around the string attached to the top of the cup); B) ‘shape’ trials (a laminated coloured shape attached to the front of the cup); and C) ‘card’ trials (a laminated coloured and/or patterned card underneath the cup). (DOCX) [file pone.0301298.s001.docx]
