# Supplementary material for: Eurasian jays (Garrulus glandarius) show episodic-like memory through the incidental encoding of information
Source: PLoS One. 2024 May 15;19(5):e0301298. doi: 10.1371/journal.pone.0301298 (PMC11095760; doi:10.1371/journal.pone.0301298)
Supplement: S1 Table — Letters (e.g., BWGY) represent the main characteristic colour (B = blue, W = white, G = green, Bk = black, P = pink, and Y = yellow) and the order (from left to right) of the cups. Underlined letters represent the baited cup and therefore the correct choice. (DOCX) [file pone.0301298.s002.docx]

**Eurasian jays (*Garrulus glandarius*) show episodic-like memory through the incidental encoding of information**

**James R. Davies, Elias Garcia-Pelegrin, and Nicola S. Clayton**

| **S1 Table.** Summary of test results showing individual choices for each trial, including trial number, trial type (shape, string, and card), the number of retraining trials (*RT*) conducted (until 5 in a row correct) and the arrangement in which the cups were presented in both the encoding phase and the memory phase. Letters (e.g., BWGY) represent the main characteristic colour (B = blue, W = white, G = green, Bk = black, P = pink, and Y = yellow) and the order (from left to right) of the cups. Underlined letters represent the baited cup and therefore the correct choice. | | | | | | | |
| --- | --- | --- | --- | --- | --- | --- | --- |
| *Bird* | *Sex* | *Trial* | *Trial type* | *RT* | *Cups (Encoding)* | *Cups (Memory)* | *Choice* |
| Godot | M | 1 | SHAPE | 5 | BkWGY | YBkGW | G |
|  |  | 2 | STRING | 5 | GBPY | BYGP | B |
|  |  | 3 | CARD | 6 | YBkWG | GYWBk | Bk |
| Sojka | F | 1 | STRING | 5 | BPGY | GPBY | G |
|  |  | 2 | CARD | 5 | YBkGW | WBkYG | W |
|  |  | 3 | SHAPE | 5 | BkYGW | BkWYG | NA |
| Jaylo | F | 1 | STRING | 5 | BGPY | PBGY | B |
|  |  | 2 | SHAPE | 8 | BkYWG | GYBkW | G |
|  |  | 3 | CARD | 5 | YWBkG | BkYGW | Bk |
| Stuka | F | 1 | SHAPE | 5 | GWBkY | GWYBk | G |
|  |  | 2 | CARD | 5 | WYGBk | BkYWG | W |
|  |  | 3 | STRING | 5 | GBYP | GBYP | B |
| Booster | M | 1 | SHAPE | 5 | WBkYG | YBkWG | Bk |
|  |  | 2 | CARD | 5 | YBkGW | BkWGY | Bk |
|  |  | 3 | STRING | 7 | YBPG | BGYP | P |
| Homer | M | 1 | CARD | 5 | GYBkW | WYBkG | G |
|  |  | 2 | STRING | 5 | PBYG | YGPB | Y |
|  |  | 3 | SHAPE | 5 | WBkYG | YGBkW | Y |
| Poe | M | 1 | CARD | 5 | WBkYG | YGWBk | Bk |
|  |  | 2 | SHAPE | 7 | BkYGW | BkYWG | W |
|  |  | 3 | STRING | 5 | BPGY | GPBY | Y |
