# Supplementary material for: Eurasian jays (Garrulus glandarius) show episodic-like memory through the incidental encoding of information
Source: PLoS One. 2024 May 15;19(5):e0301298. doi: 10.1371/journal.pone.0301298 (PMC11095760; doi:10.1371/journal.pone.0301298)
Supplement: S2 Table — Letters (e.g., BWGY) represent the main characteristic colour (B = blue, W = white, G = green, Bk = black, P = pink, and Y = yellow) and the order (from left to right) of the cups. Underlined letters represent the baited cup, and thus the correct choice according to the visual information (the cups’ visual features). (DOCX) [file pone.0301298.s003.docx]

**Eurasian jays (*Garrulus glandarius*) show episodic-like memory through the incidental encoding of information**

**James R. Davies, Elias Garcia-Pelegrin, and Nicola S. Clayton**

| **S2 Table.** Summary of control test results showing individual choices for each trial, including trial type (shape, string, and card), trial number, and the arrangement in which the cups were presented in both the initial phase and the second phase after rearranging. Letters (e.g., BWGY) represent the main characteristic colour (B = blue, W = white, G = green, Bk = black, P = pink, and Y = yellow) and the order (from left to right) of the cups. Underlined letters represent the baited cup, and thus the correct choice according to the visual information (the cups’ visual features). | | | | | | |
| --- | --- | --- | --- | --- | --- | --- |
| *Bird* | *Sex* | *Trial Type* | *Trial* | *Cups (1st)* | *Cups (2nd)* | *Choice* |
| Stuka | F | SHAPE | 1 | GYBkW | YWGBk | Bk |
|  |  |  | 2 | BkYGW | WGYBk | W |
|  |  |  | 3 | GWBkY | BkGWY | Bk |
|  |  |  | 4 | GBkYW | YGBkW | Y |
|  |  |  | 5 | YGWBk | YBkGW | W |
| Jaylo | F | STRING | 1 | BGYP | PYGB | Y |
|  |  |  | 2 | PYBG | BGPY | B |
|  |  |  | 3 | BGYP | YBGP | Y |
|  |  |  | 4 | GBPY | BGYP | Y |
|  |  |  | 5 | PBYG | PGBY | Y |
| Homer | M | CARD | 1 | WBkGY | BkWYG | G |
|  |  |  | 2 | BkYWG | WYBkG | W |
|  |  |  | 3 | YWGBk | WYBkG | W |
|  |  |  | 4 | WGYBk | WYGBk | Y |
|  |  |  | 5 | BkYWG | GYWBk | Bk |
| Poe | M | SHAPE | 1 | YGBkW | WYBkG | W |
|  |  |  | 2 | WYGBk | YBkWG | Y |
|  |  |  | 3 | YGWBk | YBkGW | Bk |
|  |  |  | 4 | BkYWG | YGBkW | Y |
|  |  |  | 5 | GYBkW | WBkYG | W |
| Sojka | F | STRING | 1 | YGBP | YBPG | B |
|  |  |  | 2 | GPBY | PBYG | B |
|  |  |  | 3 | YGPB | YPBG | G |
|  |  |  | 4 | BYGP | YPBG | Y |
|  |  |  | 5 | PYGB | GPBY | G |
| Godot | M | CARD | 1 | WYBkG | BkGYW | G |
|  |  |  | 2 | GYBkW | YBkWG | W |
|  |  |  | 3 | GWYBk | YBkGW | Y |
|  |  |  | 4 | BkYGW | BkGWY | G |
|  |  |  | 5 | GBkWY | WYGBk | Bk |
| Booster | M | SHAPE | 1 | BkGYW | GYWBk | Y |
|  |  |  | 2 | WBkYG | YWBkG | Bk |
|  |  |  | 3 | GYBkW | BkYWG | G |
|  |  |  | 4 | WYGBk | GBkWY | W |
|  |  |  | 5 | YGWBk | WGYBk | Bk |
